# Association Between C-Reactive Protein and Risk of Amyotrophic Lateral Sclerosis: A Mendelian Randomization Study

**DOI:** 10.3389/fgene.2022.919031

**Published:** 2022-05-20

**Authors:** Yahui Zhu, Mao Li, Jinghong Zhang, Xusheng Huang

**Affiliations:** ^1^ Medical School of Chinese PLA, Beijing, China; ^2^ Department of Neurology, The First Medical Center, Chinese PLA General Hospital, Beijing, China

**Keywords:** amyotrophic lateral sclerosis, mendelian randomization, C-reactive protein, single-nucleotide polymorphisms, causal relationship

## Abstract

**Background:** Until now, the relationship between C-reactive protein (CRP) levels and amyotrophic lateral sclerosis (ALS) risk has not been fully established. It is necessary to assess whether there is a causal relationship between C-reactive protein levels and ALS risk.

**Objective and Methods:** We aimed to determine whether CRP has causal effects on risk of ALS. In this present study, summary-level data for ALS (20,806 cases and 59,804 controls) was obtained from large analyses of genome-wide association studies. For instrumental variables, 37 single nucleotide polymorphisms that had been previously identified to be related to CRP levels were used, including 4 SNPs of conservative CRP genetic variants and 33 SNPs of liberal CRP genetic variants. MR estimates were calculated using the inverse-variance weighted method, supplemented by MR-Egger, weighted median, and MR-PRESSO methods.

**Results:** There was no significant causal relationship between genetically predicted CRP levels and ALS risk (OR = 1.123, 95% CI = 0.963–1.309, *p* = 0.139) and results for the conservative CRP instruments were consistent (OR = 0.964, 95% CI = 0.830–1.119, *p* = 0.628). Pleiotropic bias was not observed in this study.

**Conclusions:** This study suggests that genetically predicted CRP levels may not be a causal risk factor for ALS.

## Introduction

Amyotrophic lateral sclerosis (ALS) is a lethal neurodegenerative disease characterized by motor neuron axonal degeneration, with an average survival of 3–5 years after symptom onset (Turner et al., 2013). Serum C-reactive protein (CRP) is a biomarker of systemic inflammation (Koenig et al., 1999) and has also been considered a biomarker of neurodegeneration (Luan and Yao., 2018). The previous studies have found elevated levels of serum C-reactive protein in ALS patients (Ryberg et al., 2010) (Cui et al., 2020), but Huang et al. suggests no significant difference of CRP levels in ALS patients when compared to controls (Huang et al., 2020). Several studies have evaluated the role of CRP as a prognostic marker in ALS and the relationship between CRP and disease progression/survival rate. One study suggests that ALS patients with elevated CRP levels have faster disease progression than those with lower CRP levels and that serum CRP may be a prognostic biomarker in ALS (Lunetta et al., 2017). However, the other study showed no association of serum CRP with survival rate (De Schaepdryver et al., 2020). ALS usually occurs in middle-aged people, and this age group is often accompanied by other complications, which may affect CRP level. Since data on the association of CRP levels with ALS are often derived from observational studies, which could be subject to potential confounding bias and reverse causes, such as chronic diseases, cardiovascular risk factors and so on, it is unclear whether CRP levels are a risk factor for ALS.

Mendelian randomization (MR) is a novel method to evaluate the causal relationship between risk factors and diseases using genetic variation in observational studies (Davies et al., 2018). Due to the random assignment of genes at conception, genetic variants predate disease development and are not influenced by environmental risk factors. Thus, MR overcomes the core deficiencies of observational studies, minimizing confounding bias and reverse causality, allowing the assessment of potential causality (Walker et al., 2017).

Here, we performed a two-sample Mendelian randomization to further understand the causal effects of C-reactive protein levels on the risk of ALS. In this study, single nucleotide polymorphisms (SNPs) related to CRP levels were used as instrumental variables.

## Materials and Methods

As all analyses were performed using publicly available genome-wide association study (GWAS) summary data, no additional ethical permission was required from institutional research ethics committees. This study followed the Strengthening the Reporting of Observational Studies in Epidemiology Using Mendelian Randomization (STROBE-MR) guide (Skrivankova et al., 2021).

### Study Design

**FIGURE 1:**
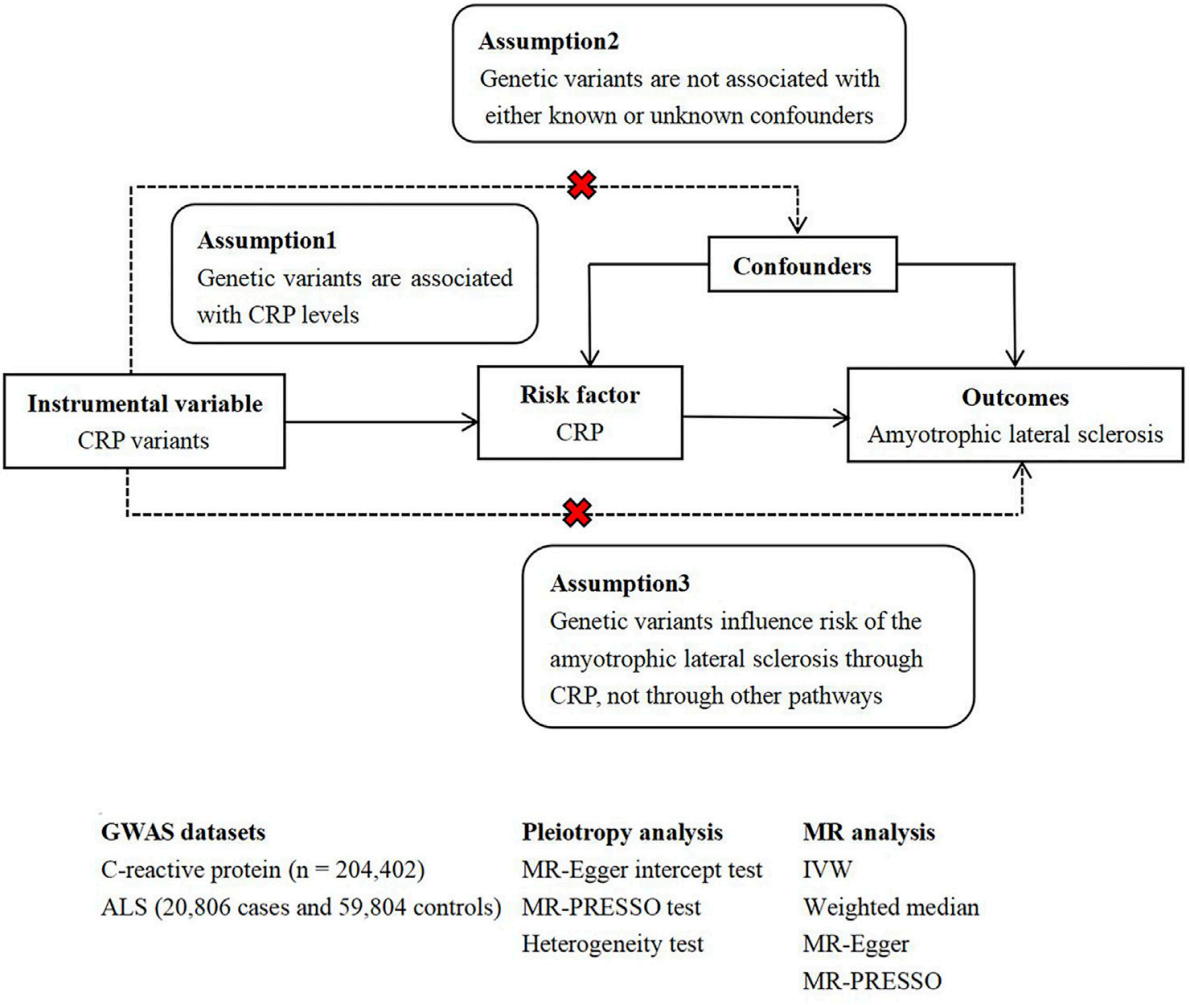
The flow chart of the MR study design.

In general, MR studies must satisfy three principal assumptions, as shown in Figure 1, which is a flow chart of our research design. Both the second and third hypotheses are designed to ensure independence from pleiotropy, as described in some previous studies (He et al., 2020) (Zhang H. et al., 2020).

### C-Reactive Protein Genetic Variants

We selected two sets of CRP genetic variants as instrumental variables, including conservative CRP genetic variants (Wensley et al., 2011) and liberal CRP genetic variants (Ligthart et al., 2018).

Wensley et al. (Wensley et al., 2011) used detailed information about the composition of the CRP gene to select a set of SNPs (rs3093077, rs1205, rs1130864, and rs1800947) that fully covers the common variations of CRP gene in populations of European descent (that is, minor allele frequency ≥0.05 and an r^2^ threshold of ≥0.8). The 4 SNPs (rs3093077, rs1130864, rs1205 and rs1800947) were at the CRP locus and used as conservative CRP instrumental variables.

Liberal CRP instrumental variables were extracted from a pooled analysis of GWAS by using data from 88 studies comprising 204,402 European individuals. The GWAS meta-analyses of CRP revealed 58 distinct genetic loci (*p* < 5 × 10^–8^). The lead variants at the distinct loci explained up to 7.0% of the variance in circulating amounts of CRP. In this study, serum CRP levels were measured using standard laboratory techniques. The authors excluded individuals with autoimmune disease, taking immunomodulators, or C-reactive protein levels four standard deviations or more from the mean. Analyses were adjusted for age, sex, population substructure, and correlation. Liberal CRP instrumental variants represented that SNPs were extracted from CRP GWAS dataset mentioned above and at the distinct genetic loci, such as C6orf173, FABP1, IL1R1 and so on.

### Amyotrophic Lateral Sclerosis Genome-Wide Association Study Dataset

The current study was based on publicly available ALS GWAS summary statistics data, including 20,806 ALS cases and 59,804 controls in people of European ancestry (Nicolas et al., 2018). In the study, Nicolas et al. undertook a large-scale GWAS involving 12,663 patients diagnosed with ALS and 53,439 control subjects and incorporated into a meta-analysis with GWAS involving 12,577 ALS cases and 23,475 control subjects (van Rheenen et al., 2016). After imputation and quality-control measures, variants from 20,806 ALS cases and 59,804 control samples were available for association analysis. All ALS patients included in the case cohort were diagnosed by neurologists specializing in ALS according to the El Escorial criteria (Brooks, 1994).

We compared the sources of participants in the CRP GWAS dataset and ALS GWAS dataset. Since the participants were from different studies or consortiums, we thought that the probability of overlapping samples between CRP GWAS and ALS GWAS was small.

### Mendelian Randomization Analysis

Conservative CRP instrumental variablesrs were rs3093077, rs1130864, rs1205 and rs1800947. For liberal CRP instrumental variables, we first identified significant SNPs(*p* < 5 × 10^–8^) related to C-reactive protein from the summary analysis of GWAS. SNPs for CRP were clumped using standard parameters (clumping window of 10000 kb, r^2^ cutoff value of 0.001) to discard variants in linkage disequilibrium (LD). Here, 57 independent SNPs were found to be associated with CRP. If SNPs were absent in the ALS GWAS dataset and for which proxies (r^2^ > 0.9) were not available by searching the online website SNiPA (http://snipa.helmholtzmuenchen.de/snipa3/), these unavailable SNPs would be excluded from downstream analysis. Subsequently, to satisfy the second assumption, we used the PhenoScanner tool (Kamat et al., 2019) to examine whether selected SNPs were associated with potential confounders affecting ALS. When using the PhenoScanner tool, the threshold for genome-wide significance was set at *p* < 5 × 10^–8^. In addition, we applied MR Steiger filtering (Hemani et al., 2017) to test the causal direction of the obtained SNPs on exposures and outcomes. We excluded SNPs with “FALSE” results because these SNPs mainly affected the outcomes, not exposures. Finally, we assessed the power of remaining SNPs using the F statistics (F = beta^2^/se^2^) for each SNP. SNPs with less statistical power would be removed to avoid weak instrumental variables (F statistics <10) (Chen L. et al., 2021).

Pleiotropy analyses were mainly based on three different statistical methods, including the MR-Egger intercept test (Verbanck et al., 2018), MR Pleiotropy RESidual Sum and Outlier (MR-PRESSO) global test (Verbanck et al., 2018), and the heterogeneity test using Cochran’s Q statistic (Greco et al., 2015). Statistically significant differences for the above analyses were set at *p* value < 0.05. In addition, we depicted funnel plots to visualize any heterogeneity of effect estimates. Asymmetry about the vertical line is indicative of the heterogeneity.

Six MR analysis methods were selected including the inverse-variance weighted (IVW), weighted median, MR-Egger, MR-PRESSO, simple mode, and weighted mode test. The random-effects IVW method, the main method of the study, essentially assumed a zero intercept and performed a weighted regression of the SNP-exposure effects with the SNP-outcome effects. The MR Egger method provided more conservative causal estimates in the presence of pleiotropy and was less likely to produce exaggerated test statistics (Burgess and Thompson, 2017). Even when up to 50% of the information in the analysis came from invalid IVs, the weighted median method could provide valid estimates (Bowden et al., 2016). The MR-PRESSO method was used to detect outliers that might bias the results and to assess whether causal estimates change after removing outliers (Verbanck et al., 2018). In addition, we performed a leave-one-SNP-out analysis. In this analysis, we systematically removed one SNP at a time, assessing the impact of potentially pleiotropic SNPs on causal effects. Estimates were expressed as odds ratio (OR) and 95% confidence interval (CI) per unit increase in natural log-transformed genetically predicted CRP levels (mg/L). Statistical analysis was performed in version R4.1.2 (TwoSampleMR and MR-PRESSO packages). The signifcance threshold was *p* value < 0.05.

### Power Analysis

The proportion of CRP variance was explained by each instrument SNP R^2^, which was calculated using the following formula:R^2^ = 2β^2^MAF (1–MAF) (Park et al., 2010). Where MAF represents the minor allele frequency of the instrument SNP, and β denotes the effect size for SNP. The statistical power was calculated using the web-based tool mRnd, where the two-sided type-I error rate α was 0.05 (Brion et al., 2013).

## Results

### Conservative CRP Genetic Variants With Amyotrophic Lateral Sclerosis

**FIGURE 2:**
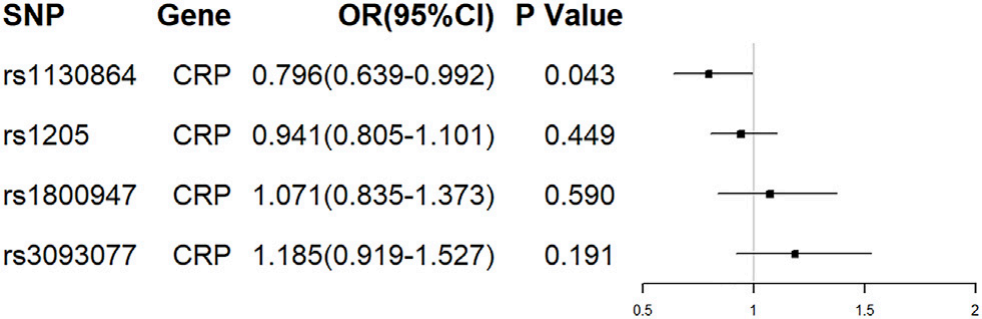
The effect of single conservative CRP genetic variants on ALS. OR, odds ratio; CI, confidence interval.

**FIGURE 3:**
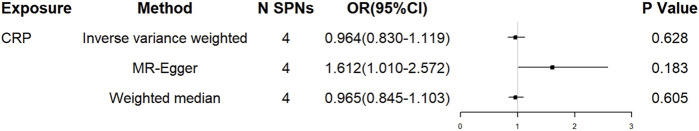
The effect of overall conservative CRP genetic variants on ALS. OR, odds ratio; CI, confidence interval.

Of the 4 SNPs, only 1 was shown to be significantly associated with the risk of ALS (OR = 0.796, 95% CI = 0.639–0.992, *p* = 0.043) and the other 3 SNPs were showed no association with the risk of ALS (Figure 2). Overall, there was no evidence to suggest a causal association between CRP levels and ALS risk in the analysis using IVW method with an OR of 0.964 (95% CI = 0.830–1.119, *p* = 0.628). The results were consistent in the analyses using weighted median and MR Egger methods (Figure 3). The detailed characteristics of the 4 SNPs was showed in Table 1.

**TABLE 1:** The characteristics of the selected conservative CRP instrumental variables.

SNP	Effect Allele	Other Allele	Association with CRP			Amyotrophic Lateral Sclerosis		
			EAF	β	SE	β	SE	P
rs3093077	C	A	0.0716	0.21	0.018	0.0356	0.0272	0.1913
rs1205	C	T	0.6899	0.18	0.01	−0.0109	0.0144	0.4501
rs1130864	A	G	0.327	0.13	0.008	−0.0296	0.0146	0.04302
rs1800947	C	G	0.9429	0.26	0.015	0.0178	0.033	0.5905

SNPs, single nucleotide polymorphisms; EAF, effect allele frequency; SE, standard error.

Heterogeneity was not observed using Cochran Q statistic based on IVW (*p* = 0.102) and MR-Egger (*p* = 0.519). The MR Egger intercept (intercept = -0.092, SE = 0.042, *p* = 0.158) showed no horizontal pleiotropy. In PhenoScanner database, none of the 4 SNPS were observed to be associated with other traits, diseases or risk factors.

### Liberal CRP Genetic Variants With Amyotrophic Lateral Sclerosis

A total of 57 independent SNPs were found to be associated with CRP. Rs644234 was excluded in downstream analysis because it was not available in ALS data. Since rs2794520 was at the CRP locus, in order to reduce horizontal pleiotropy, we would analyze the effect of rs2794520 and other 55 SNPs on ALS risk separately.

Rs2794520 explained 1.48% variance of CRP. For rs2794520, we did not observe a causal relationship between CRP levels and ALS risk (OR = 0.949, 95% CI = 0.813–1.108, *p* = 0.505).

For the 55 SNPs, harmonising CRP and ALS, the following SNPs for being palindromic with intermediate allele frequencies were excluded: rs10778215 and rs11108056. When using the PhenoScanner tool, we excluded 20 SNPs that were associated with confounders, which were proved to be causally associated with ALS such as low density lipoprotein (LDL), total cholesterol (Chen et al., 2018), type 2 diabetes (T2DM) (Chen H. et al., 2021), childhood body mass index (Zhang L. et al., 2020), neutrophil count, white blood cell count (Li et al., 2020), systolic blood pressure and diastolic blood pressure (Xia et al., 2022). The remaining SNPs were all with true causal direction identified by the MR Steiger filtering. The F statistics for each SNP were greater than the statistical threshold of 10, indicating sufficient validity for all SNPs. Thus, the MR analysis of ALS included 33 SNPs related to CRP. The detailed characteristics of the SNPs was showed in Supplementary Table S1. All these 33 genetic variants could explain 1.55% variance of CRP.

**FIGURE 4:**
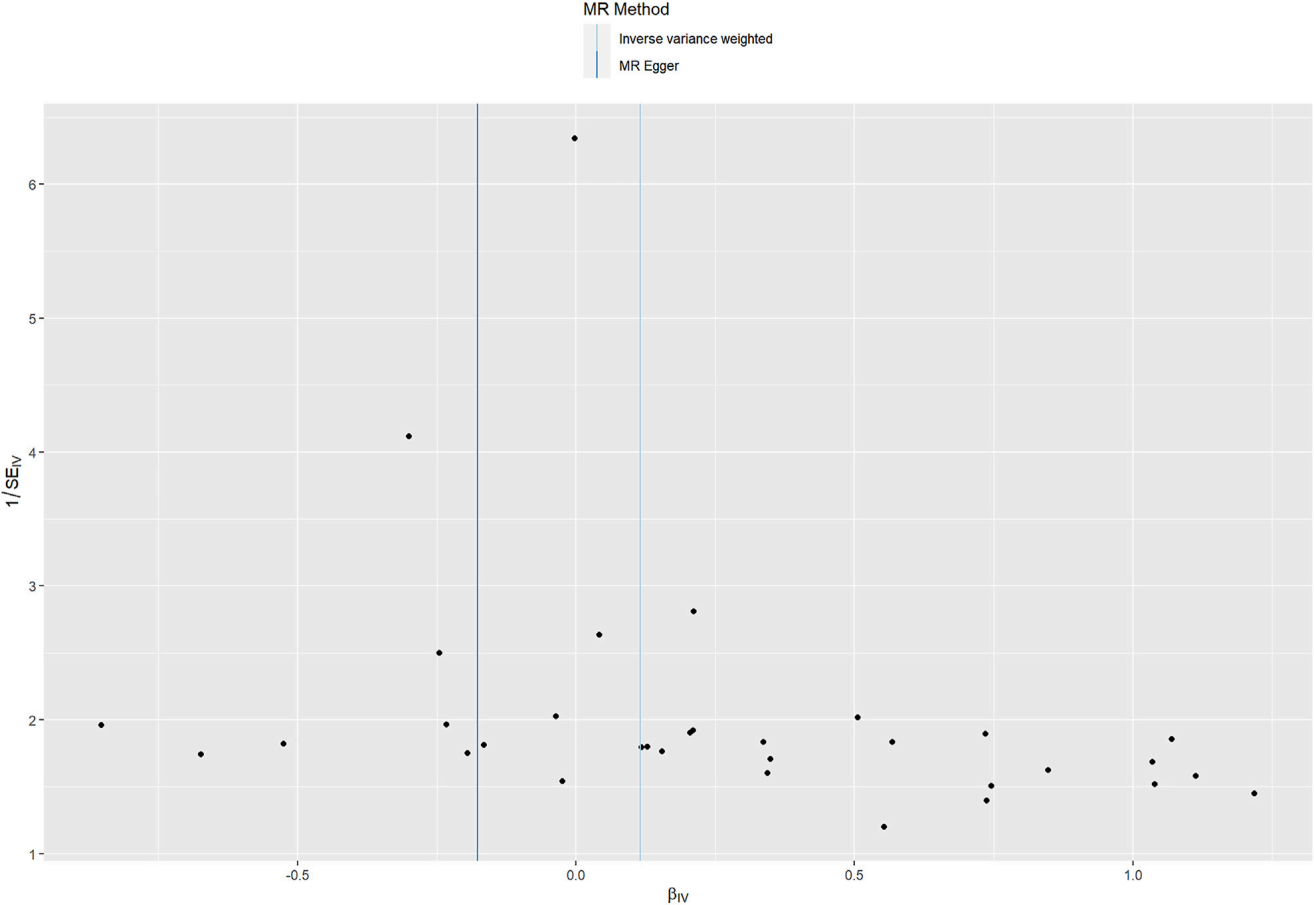
Funnel plot assessing heterogeneity. Blue line represents the inverse-variance weighted estimate, and dark blue line represents the MR-Egger estimate. SE, standard error; IV, instrumental variable.

Cochran Q statistic based on IVW (*p* = 0.491) and MR-Egger (*p* = 0.613) showed no evidence of heterogeneity and the symmetry of the funnel plot supported that (Figure 4). The MR Egger intercept (intercept = 0.011, SE = 0.006, *p* = 0.077) suggested no horizontal pleiotropy for instrumental variables, and the MR-PRESSO global test (*p* = 0.482) supported that. The MR-PRESSO outlier test did not identify outlier SNPs. Therefore, the selected 33 SNPs associated with CRP were used as instrumental variables for the downstream MR analysis.

**FIGURE 5:**
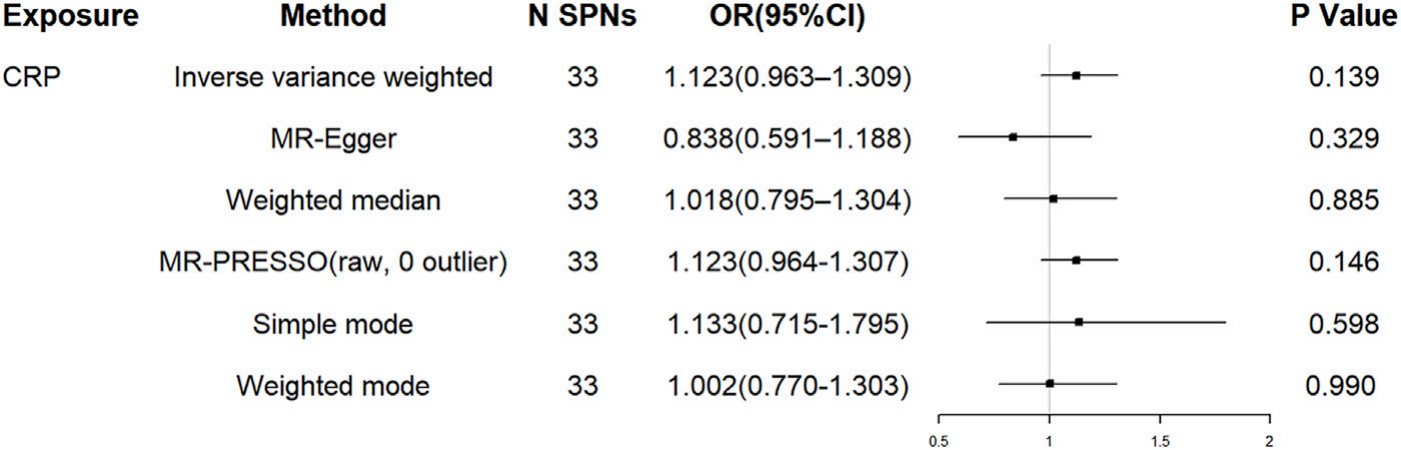
The effect of liberal CRP genetic variants on ALS. OR, odds ratio; CI, confidence interval; MR-PRESSO, MR Pleiotropy RESidual Sum and Outlier.

**FIGURE 6:**
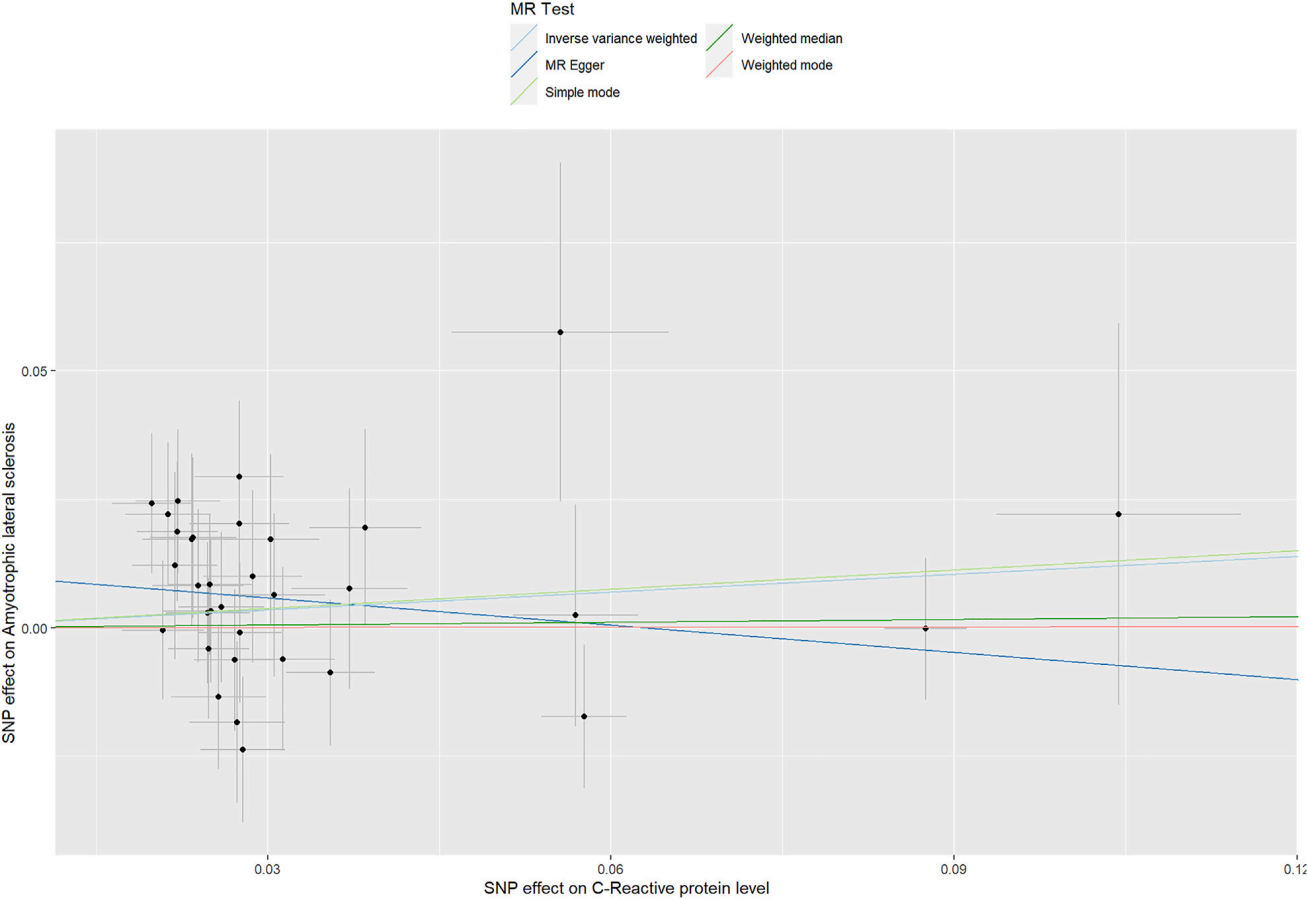
Scatter plots of genetic associations of CRP with ALS risk. The slopes of each line represent the causal association for each method.

**FIGURE 7:**
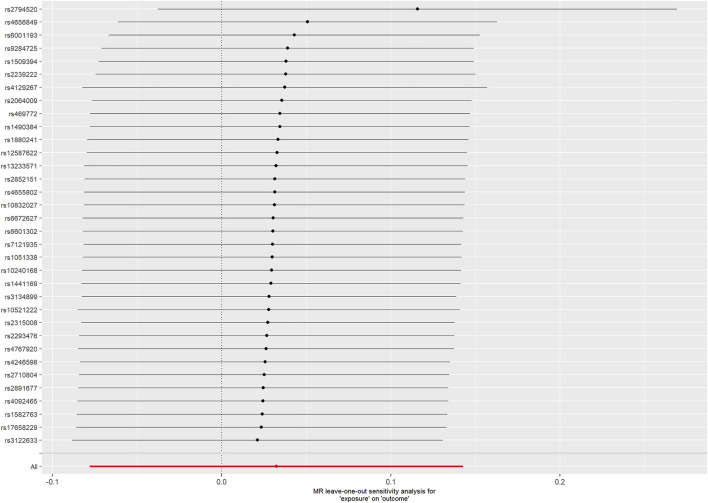
MR leave-one-out sensitivity analysis for CRP on ALS.

For the 33 SNPs, there was no evidence of a causal relationship between genetically predicted CRP levels and ALS risk, with *p* values >0.05 in the analysis using IVW approach with an OR of 1.123 (95% CI = 0.963–1.309, *p* = 0.139). The other analyses, MR Egger (OR = 0.838, 95% CI = 0.591–1.188, *p* = 0.329), weighted median (OR = 1.018, 95% CI = 0.795–1.304, *p* = 0.885) and MR-PRESSO (OR = 1.123, 95% CI = 0.964–1.307, *p* = 0.146) methods supported these results (Table 2; Figure 5 and Figure 6). In the leave-one-out analysis, we did not observe a single SNP of CRP to have an influence on the association (Figure 7).

**TABLE 2:** The causal association of CRP levels with ALS risk.

Method	N SNPs	OR	95%CI	*p* Value
IVW	33	1.123	0.963–1.309	0.139
MR-Egger	33	0.838	0.591–1.188	0.329
Weighted median	33	1.018	0.795–1.304	0.885
MR-PRESSO (raw, 0 outlier)	33	1.123	0.964–1.307	0.146
simple mode	33	1.133	0.715–1.795	0.598
weighted mode	33	1.002	0.770–1.303	0.990

SNPs, single nucleotide polymorphisms; OR, odds ratio; CI, confidence interval; IVW, inverse-variance weighted; MR-PRESSO, MR, Pleiotropy RESidual Sum and Outlier.

### Power Analysis

For liberal CRP genetic variants with ALS, our MR study had 80% power to detect an OR of 1.191 or higher per SD increase in C-reactive protein levels for ALS with an alpha of 5%.

## Discussion

Until now, the relationship between C-reactive protein levels and ALS risk has not been fully established. Hence, it is necessary to clarify the causal relationship between CRP levels and ALS risk in order to develop effective therapeutic and preventive measures. In our study, we selected 4 SNPs and 33 SNPs as conservative CRP instrumental variables and liberal CRP instrumental variables and obtained their corresponding summary statistics in the ALS GWAS dataset. After that, we evaluated the causal link of CRP levels with ALS risk by MR analysis. Our study showed no causal effects between CRP levels and ALS risk. CRP levels might not increase or reduce the risk of ALS.

Inflammation is involved in the pathogenesis of central nervous system neurodegenerative diseases, including ALS (Stephenson et al., 2018). The neuroinflammation of ALS is predominantly characterized by activation of microglia and astrocytes innate immune sensing pathways to the central nervous system (McCauley and Baloh, 2019). CRP, as a biomarker of low-grade inflammation, has been proposed to play a role in the development of ALS. However, a recent study showed that compared to controls, patients with ALS had lower CRP levels before diagnosis until 1 year after diagnosis. After that, ALS patients had higher CRP levels when compared to controls. This study suggested that C-reactive protein was involved in the disease course of ALS, but perhaps only in the later disease stages (Cui et al., 2020). Therefore, this study also supports our findings to a certain extent. That is, C-reactive protein may not be a risk factor for the development of ALS. Although persistent inflammation is thought to be a contributor in the development of ALS, inflammation may promote the development of ALS through inflammatory mediators other than C-reactive protein. Hence, there is no clear causal relationship between C-reactive protein levels and ALS risk.

One of the advantages of our study is that the use of MR analysis reduces potential confounding factors and reverse causality, minimizing bias from the traditional observational studies. To our knowledge, this is the first study to investigate the causal relationship between CRP and ALS risk using genetic variation. In addition, the large sample size of this study and the inclusion of multiple SNPs as instrumental variables improve statistical power, which increases the ability to identify weak associations. Finally, no pleiotropy is observed in this study, and these models produce similar conclusions, suggesting the robustness of the findings.

Our study also has some limitations. First, since this study used summary-level data, the possibility of a non-linear association between CRP levels and the risk of ALS cannot be completely ruled out. Second, as this study was based on pooled-level data, we could not conduct a more detailed subgroup analysis. Therefore, in further studies, the opportunity to obtain more detailed data on the individual-level, such as patient’s age, gender, and so on, will help us further understand the causal relationship between CRP levels and the risk of ALS in each subgroup. We look forward to the opportunity for authors of the publicly available GWAS to share individual-level data for further research. Third, bi-directional MR analysis was not carried out. For ALS as exposure, we identified significant SNPs (*p* < 5 × 10^–8^) associated with ALS from the summary analysis of GWAS. Then, SNPs for ALS were clumped using standard parameters (clumping window of 10000 kb, r^2^ cutoff value of 0.001) to discard variants in linkage disequilibrium (LD). Here, 6 independent SNPs associated with ALS were found. However, only one SNP (rs3849938) in the CRP (outcome) GWAS dataset was available. We considered that the number of instrumental variables was too small for MR analysis, so bi-directional MR analysis was not carried out. In addition, since all participants (including CRP levels and ALS) are of European ancestry, this study may not be available in other ethnic groups, making it difficult to extrapolate the research results to other ancestries. Hence, the findings should be further replicated in other ancestries.

To conclude, we found no evidence to support a causal relationship between CRP levels and the risk of ALS. In the future, we expect a larger GWAS database and individual-level data to be available. Meanwhile, additional studies are also expected to further confirm our findings.

## Data Availability

Publicly available datasets were analyzed in this study. This data can be found here: https://gwas.mrcieu.ac.uk/.
