# Supplementary material for: Association Between C-Reactive Protein and Risk of Amyotrophic Lateral Sclerosis: A Mendelian Randomization Study
Source: Front Genet. 2022 May 20;13:919031. doi: 10.3389/fgene.2022.919031 (PMC9164009; doi:10.3389/fgene.2022.919031)
Supplement: Supplementary file 1 [file Table1.DOC]

Supplementary Table 1 The characteristics of the selected liberal CRP instrumental variables

| SNP | Effect allele | Other allele | Association with CRP | | | Amyotrophic lateral sclerosis | | |
| --- | --- | --- | --- | --- | --- | --- | --- | --- |
|  |  |  | EAF | β | SE | β | SE | P |
| rs10240168 | G | C | 0.221389 | -0.028684 | 0.004343 | -0.01 | 0.0168 | 0.552199 |
| rs1051338 | G | T | 0.308119 | 0.023881 | 0.003992 | 0.0082 | 0.0149 | 0.583201 |
| rs10521222 | T | C | 0.04533 | -0.104411 | 0.010714 | -0.0221 | 0.0372 | 0.5525 |
| rs10832027 | A | G | 0.668809 | 0.025944 | 0.003745 | 0.004 | 0.0147 | 0.7867 |
| rs12587622 | A | G | 0.500861 | -0.020798 | 0.003609 | 5.00E-04 | 0.0135 | 0.9727 |
| rs13233571 | T | C | 0.119609 | -0.056895 | 0.005476 | -0.0024 | 0.0216 | 0.9113 |
| rs1441169 | G | A | 0.532568 | -0.024926 | 0.003725 | -0.0084 | 0.0136 | 0.539 |
| rs1490384 | T | C | 0.512283 | -0.024816 | 0.003545 | 0.0041 | 0.0137 | 0.7618 |
| rs1509394 | T | C | 0.542959 | 0.025685 | 0.004147 | -0.0135 | 0.0141 | 0.3379 |
| rs1582763 | A | G | 0.37181 | -0.022107 | 0.0037 | -0.0246 | 0.014 | 0.07993 |
| rs17658229 | C | T | 0.045881 | 0.055568 | 0.009522 | 0.0575 | 0.033 | 0.0812606 |
| rs1880241 | G | A | 0.48295 | -0.027537 | 0.003687 | 0.001 | 0.0136 | 0.9432 |
| rs2064009 | T | C | 0.581445 | 0.027111 | 0.003549 | -0.0063 | 0.0138 | 0.6474 |
| rs2239222 | G | A | 0.363541 | 0.035484 | 0.003901 | -0.0087 | 0.0142 | 0.5434 |
| rs2293476 | C | G | 0.225941 | 0.030262 | 0.004225 | 0.0172 | 0.0165 | 0.2954 |
| rs2315008 | G | T | 0.687731 | 0.023467 | 0.003777 | 0.0175 | 0.0156 | 0.2631 |
| rs2710804 | C | T | 0.367409 | 0.021262 | 0.003737 | 0.0221 | 0.014 | 0.1148 |
| rs2852151 | A | G | 0.404527 | 0.024735 | 0.003655 | 0.0029 | 0.0138 | 0.8357 |
| rs2891677 | T | C | 0.538958 | 0.019859 | 0.003511 | 0.0242 | 0.0137 | 0.0777302 |
| rs3122633 | C | T | 0.301705 | 0.027479 | 0.00389 | 0.0294 | 0.0148 | 0.04701 |
| rs3134899 | T | C | 0.767055 | 0.023329 | 0.004274 | 0.0172 | 0.0167 | 0.3012 |
| rs4092465 | G | A | 0.650698 | 0.027483 | 0.004364 | 0.0202 | 0.0145 | 0.1626 |
| rs4129267 | T | C | 0.385524 | -0.087519 | 0.003612 | 2.00E-04 | 0.0138 | 0.9889 |
| rs4246598 | A | C | 0.46258 | 0.022063 | 0.003547 | 0.0187 | 0.0136 | 0.1691 |
| rs4655802 | A | G | 0.614793 | -0.025012 | 0.00416 | -0.0032 | 0.0139 | 0.8199 |
| rs4656849 | G | A | 0.621343 | 0.057656 | 0.003723 | -0.0173 | 0.014 | 0.2163 |
| rs469772 | T | C | 0.193541 | -0.031327 | 0.004542 | 0.0061 | 0.0179 | 0.7331 |
| rs4767920 | A | G | 0.842127 | -0.038519 | 0.0049 | -0.0195 | 0.0191 | 0.3053 |
| rs6001193 | G | A | 0.35065 | -0.027809 | 0.003706 | 0.0237 | 0.0142 | 0.0940091 |
| rs6601302 | G | T | 0.736931 | -0.030518 | 0.004478 | -0.0064 | 0.0159 | 0.687401 |
| rs6672627 | A | C | 0.146148 | -0.037135 | 0.005083 | -0.0076 | 0.0195 | 0.6957 |
| rs7121935 | A | G | 0.376337 | -0.021853 | 0.00374 | -0.0121 | 0.0182 | 0.5062 |
| rs9284725 | A | C | 0.761212 | -0.02731 | 0.00419 | 0.0184 | 0.0157 | 0.2412 |
| rs2794520 | T | C | 0.333842 | -0.182186 | 0.003712 | 0.0096 | 0.0144 | 0.505 |

SNPs, single nucleotide polymorphisms; EAF, effect allele frequency; SE, standard error.
